# Undertaking Rehabilitation Research During the COVID-19 Pandemic: Emergent Strategies From a Trainee-Faculty Workshop

**DOI:** 10.3389/fresc.2022.881606

**Published:** 2022-05-16

**Authors:** Kenneth S. Noguchi, Linda Nguyen, Ava Mehdipour, Elise Wiley, Stephanie Saunders, Kevin Moncion, Julie C. Reid, Nora Bakaa, Laura Garcia Diaz, Jill Van Damme, Cassandra D'Amore, Anne Kumurenzi, Ze Lu, Erin Knobl, Marla K. Beauchamp, Luciana G. Macedo, Brenda Vrkljan, Sandra E. Moll, Lisa C. Carlesso, Lori J. Letts, Michelle E. Kho, Julie Richardson

**Affiliations:** School of Rehabilitation Science, McMaster University, Hamilton, ON, Canada

**Keywords:** rehabilitation research, COVID-19, research methods, multidisciplinary, workshop discussion

## Abstract

**Background:**

The COVID-19 pandemic has disrupted everyday rehabilitation research. Many academic institutions have halted in-person human research including rehabilitation sciences. Researchers are faced with several barriers to continuing their research programs. The purpose of this perspective article is to report the results of an interdisciplinary workshop aimed at understanding the challenges and corresponding strategies for conducting rehabilitation research during the COVID-19 pandemic.

**Methods:**

Twenty-five rehabilitation researchers (17 trainees and eight faculty) attended a 2-h facilitated online workshop in to discuss challenges and strategies they had experienced and employed to conduct rehabilitation research during the COVID-19 pandemic.

**Results:**

Rehabilitation researchers reported challenges with (1) *pandemic protocol adjustments*, (2) *participant accessibility*, and (3) *knowledge dissemination*, along with corresponding strategies to these challenges. Researchers experienced disruptions in study outcomes and intervention protocols to adhere to public health guidelines and have suggested implementing novel virtual approaches and study toolkits to facilitate offsite assessment. Participant accessibility could be improved by engaging community stakeholders in protocol revisions to ensure equity, safety, and feasibility. Researchers also experienced barriers to virtual conferences and publication, suggested opportunities for smaller networking events, and revisiting timeframes for knowledge dissemination.

**Conclusion:**

This perspective article served as a catalyst for discussion among rehabilitation researchers to identify novel and creative approaches that address the complexities of conducting rehabilitation research during the COVID-19 pandemic and beyond.

## Introduction

Early in the COVID-19 pandemic, many academic institutions have mandated a pause on research involving populations without COVID-19, particularly in outpatient and community settings. The development of vaccines for COVID-19 (1–3) has initiated a global recovery from the pandemic, but considering the many emerging variants of the virus, public health measures have remained in place and will continue to impact human research for the foreseeable future. Despite the fluctuating enforcement of public health measures, such precautions are likely to continue to present significant barriers to conducting and disseminating rehabilitation research in Canada and beyond. It is therefore imperative that rehabilitation researchers in acute, community and long-term care settings plan for alternative approaches to continuing rehabilitation research, as we wait to resume in-person activities.

There has been much scholarly discourse on the challenges of the pandemic within the broader scientific community. A recent review discussed the difficulties in research funding, publishing, and presenting at academic conferences (4), while other papers have outlined strategies for conducting hospital-based, critical care research with COVID-19 restrictions (5). Additionally, an interdisciplinary collaboration model for rehabilitation research has been developed (6) and the challenges of clinical research faced during the pandemic have also been discussed (7). However, discussions have been limited to specific areas of rehabilitation research such as multiple sclerosis and quality of life research, and few have offered solutions to such challenges, including the perspectives of trainees.

This article reports the discussions of a facilitated online workshop, designed to engage discussion among a diverse group of trainees and faculty on the challenges and corresponding solutions for conducting rehabilitation research during the COVID-19 pandemic. The specific objectives of this paper are to (8) highlight the challenges rehabilitation researchers have faced during the global pandemic, and (1) to discuss innovative strategies that can be employed to support ongoing and future rehabilitation research under such challenging circumstances.

## Materials and Methods

The workshop was developed and delivered by rehabilitation researchers in the School of Rehabilitation Science at McMaster University in Hamilton, Ontario, Canada. The core group consisted of four PhD students (KSN, LN, AM & EW) and the Assistant Dean of the program (JR). The first author (KSN) and senior author (JR) identified the broad objectives of the facilitated workshop. Three PhD students (LN, AM, & EW) helped the first and senior authors refine the workshop's guiding questions. These students were selected for their leadership within the department, as well as for their diverse research training backgrounds, spanning quantitative and qualitative expertise in childhood disability, psychometric evaluation of measurement tools, stroke rehabilitation, and sex and gender research. The list of questions was generated by consensus *via* group discussion, considering priority areas of discussion for trainees and faculty.

The five specific guiding questions for the workshop aimed to generate reflection on the challenges for ongoing and future studies in different phases of research. The questions asked: (1) “*What are some challenges that you have faced in your research since the COVID-19 pandemic began?*”, (2) “*When COVID-19 lockdown restrictions first took place in March 2020, what were some considerations that you had to take into account for your research?*”, (3) “*What are some strategies that you have used to continue to conduct your research?”*, (4) “*For research studies that have data results, what are some challenges that you anticipate you might have with analysis?”*, and (5) “*As you move forward with your research studies, what are some plans that you might have for the knowledge translation/dissemination of your study findings?”*.

All research trainees and faculty members in the School of Rehabilitation Science at McMaster University were invited by e-mail to attend the 2-h online workshop held on commercially available video conferencing software (Zoom Video Communications, San Jose, CA, USA) in November 2020. The discussion was facilitated by authors KN and LN, who posed each guiding question to the group. Workshop attendees were able to actively participate in the discussion verbally or by use of the chat box function. The recording from the workshop was transcribed verbatim. Trainees and faculty members provided verbal consent for the workshop to be recorded and were informed that a publication was planned from the workshop findings which they were invited to co-author. Active workshop participants (i.e., those speaking or typing during the discussion) were also invited as co-authors on the manuscript, where they were able to clarify or expand on topics discussed in the workshop. All participants consented to be a co-author and reviewed the paper and provided feedback. This project did not require ethics review or approval according to the Hamilton Integrated Research Ethics Board and Tri-Council Policy Statement (TCPS-2, 2018, Article 2.5).

## Results

One-hundred and eight individuals received an invitation to participate in the facilitated workshop (92 trainees and 16 faculty). Thirty-nine attended the workshop, from which 25 actively participated (21 women, four men). Among active participants, there were 17 trainees (14 doctoral, two Master's, and one post-doctoral fellow) and eight research faculty (five physiotherapy and three occupational therapy). Three key research challenges and corresponding strategies emerged, including (1) *pandemic protocol adjustments*, (2) *participant accessibility*, and (3) *knowledge dissemination*. The following sections summarize a narrative synthesis of the workshop discussions, and are summarized in Table 1.

**Table 1:** Summary of workshop findings.

**Pandemic Protocol Adjustments**	
How can I keep collecting primary data?	• Research “toolkits” containing equipment needed for assessments • Consider community spaces to distribute study supplies • Use of wearable sensors for virtual assessment
What should I consider when collecting primary data?	• Use of focus groups with community members to understand the feasibility of data collection • Use of covariates and subgroup analyses based on participants' history of COVID-19 • Adjust for other pandemic-related contextual factors
Should I shift my in-person intervention to a virtual intervention?	• Consider the implications for intervention fidelity, safety and feasibility in participants' homes • Additional supervision in the home by a family member, when possible • Consider disparities in accessibility to virtual interventions • Consider pausing the trial until in-person activities have been permitted
**Participant Accessibility**	
How can I keep my participants safe and engaged in virtually-delivered research?	• Consider regular follow-ups with study participants • Consult with community stakeholders about the frequency of follow-ups • Consider providing detailed descriptions of privacy and safety procedures • Consider including family members to monitor safety during performance-based assessments • If safety is compromised in the virtual setting, consider temporarily suspending the trial until in-person activities resume
What if I can't recruit participants?	• Consider alternative study designs that are feasible in a remote environment
**Knowledge Dissemination**	
Should I plan to attend virtual conferences?	• Consider the additional time commitment required for virtual conferences • Conference organizers should consider implementing networking events to facilitate collaboration
What are the implications for my study's findings?	• Consider the impact of the pandemic on your study's population demographics • Consider contextualizing study findings to the global pandemic • Plan for delays in peer-review times for knowledge translation plans

### PART 1: Pandemic Protocol Adjustments

#### Challenge #1: How Can I Keep Collecting Primary Data?

Researchers shared how they often collected their physical or physiological data through in-person visits to the home or research space prior to the pandemic. The use of specialized equipment may not be feasible when adhering to public health and institutional guidelines. For example, the use of electromyography for individuals with stroke is difficult to implement in the community setting due to the cost and the logistics of transporting this equipment in participants' homes.

##### Proposed Strategies

Several researchers highlighted that certain performance-based measures, qualitative interviews, and questionnaires may still be safe and feasible in the home setting. When institutional and public health guidelines permitted, some researchers utilized existing community spaces, such as recreation centers and libraries, to recruit and distribute study materials and equipment for participants. This research group used virtual “toolkits” as an alternative to in-person assessments, which has been a viable method for continuing research studies and adhering to physical distancing guidelines. Virtual toolkits contained equipment needed for in-person assessments such as pylons, stopwatches, and instructions. A research concierge was responsible for delivering assessments virtually, through a video conferencing software. Participants are guided through the software to set up their home environment for performance-based assessments such as gait speed or balance assessments. Participants performed the task on a video conferencing software, where study staff monitor set-up and evaluate assessments.

#### Challenge #2: What Should I Consider When Collecting Primary Data?

Researchers noted that adding virtual assessments may be particularly challenging for study participants and investigators. Adding virtual assessments could restrict participation to select groups, increase the risk of selection bias and attrition in remotely delivered studies, particularly for groups with limited accessibility to internet. Researchers emphasized that adding virtually administered outcomes could also place more burden on participants, impact safety and influence the validity of their results.

In the context of clinical research, participants' history of COVID-19 can also impact several biological and/or psychosocial outcomes, thus impacting study findings. There are also many contextual and societal impacts of the COVID-19 pandemic on rehabilitation outcomes that are not explained by a history of COVID-19 (9). Closures of public recreation spaces may disproportionately affect the ability of some individuals to participate in community programs and physical activities, resulting in increased sedentary behavior.

##### Proposed Strategies

To bridge the gaps in primary data collection procedures, small focus groups may be held to incorporate participant and community members' perspectives on best methods to collect data to mitigate these challenges in specific groups. Focus groups can help researchers understand the strategies necessary to safely and equitably respond to rapidly changing guidelines. From the study staff perspective, budgeting for internet access in project grants for virtually-delivered research can also help mitigate the disparities in data collection.

Certain researchers also discussed accessing additional health information to provide context for research continuing during the pandemic. For example, they have amended ethics applications to collect information on participants' history of COVID-19 due to the potential long-term health implications of the condition has been instituted. Other researchers have used history of COVID-19 as a covariate within planned analyses and examining subgroups. Even without a COVID-19 diagnosis, researchers have opted to collect additional demographic variables such as physical activity levels or mental health assessments that may help to contextualize study findings in the pandemic.

#### Challenge #3: Should I Shift My In-Person Intervention to a Virtual Intervention?

Workshop participants identified several methodological considerations before shifting to virtual interventions. Shifting to a virtually-delivered version of an intervention that was originally intended to be in-person could have unintended methodological implications. For instance, some exercise modalities (e.g., cycle ergometry) are not always safe or feasible in a virtual format due to a lack of direct supervision or equipment in participants' homes. The participant could be supervised by another person such as a family member while exercising, however, having another trained person present is not always feasible or ethical. Additionally, environmental constraints, such as space limitations may limit participants' options in the home setting. These limitations of home-based or virtual rehabilitation may contribute to the low levels of engagement in rehabilitation, especially in higher risk populations (10). Thus, shifting to a virtual format, where participants may not have the necessary supervision, equipment, and setting to engage in rehabilitation interventions, may result in substantial variability in exposure to allocated interventions, as conditions are difficult to standardize within the participants' homes.

##### Proposed Strategies

Researchers considered which core aspects of the intervention are being impacted in the switch to virtual delivery to ensure treatment fidelity. Many researchers either terminated or paused the study when the adapted intervention produced substantial variability in participants' treatment exposure, until they can continue in-person activities. Adaptation of study interventions and designs to be easily accessible for study participants of all socioeconomic status was also necessary. Shifts from in-person to virtual format such as qualitative interviews, home-based education, and self-management interventions may transition to a virtual format preserving the fidelity of the intervention. However, congruence between methodologies performed in-person and virtual protocols needs careful consideration.

### PART 2: Participant Accessibility

#### Challenge #1: How Can I Keep My Participants Safe and Engaged in Virtually-Delivered Research?

Participants in rehabilitation studies are often older adults with chronic conditions who are disproportionately at greater risk for COVID-19 and COVID-19-related mortality (11). Therefore, older adults and immunocompromised individuals who adhere to public health physical distancing often result in increased rates of social isolation and mental health challenges (12). Safe strategies are needed to increase engagement with these participants in virtually-delivered research, without also increasing study burden.

##### Proposed Strategies

Workshop participants reported that contacting socially isolated participants to participate in virtually delivered research studies, offers an opportunity to connect and interact with study staff. Evidence suggests that more frequent interactions and follow-ups may improve participant adherence to study interventions (13). However, consultation with community stakeholders and participants about follow-up frequency of follow-ups is important to prevent burden on study participants.

To help reduce the potential burden of virtual interventions on participants, intervention procedures planned during a global pandemic may also aim to be safe, simple, and affordable. For example, some researchers suggested that video conferencing software can assist in ensuring the safe and ethical provision of informed consent. They also suggested providing detailed descriptions of safety procedures in ethics applications and study protocols to mitigate the risks of these forms of delivery. For example, the use of virtual private networks and optimized privacy settings during interactions, or detailed description of how outcomes assessment will be undertaken to mitigate concerns about falling. Therefore, allocating sufficient funding for resources and time to ensure participant safety and adherence will be important. Otherwise placing the study on hold until in-person activities resume may be necessary.

#### Challenge #2: What If I Can't Recruit Participants?

Throughout the COVID-19 pandemic, in-person rehabilitation research has been strictly prohibited in some settings, which frequently involves older adults or individuals with chronic conditions. Workshop participants noted that local hospitals allowed research on-site when patients were attending a medical appointment, but did not allow hospital visits solely for research. Researchers were challenged with the implications of the pandemic on their access to study participants. Even when research in hospitals was permitted to continue, hospitals are often operating at near-capacity due to the resurgence of COVID-19 hospitalizations (14). Many elective treatments have ceased, which has led to reduced access to participants for studies (15). As a result, there may be serious implications for feasibility of prospective and successful grant applications, where extra time is needed to achieve the target sample size.

##### Proposed Strategies

**Figure 1:**
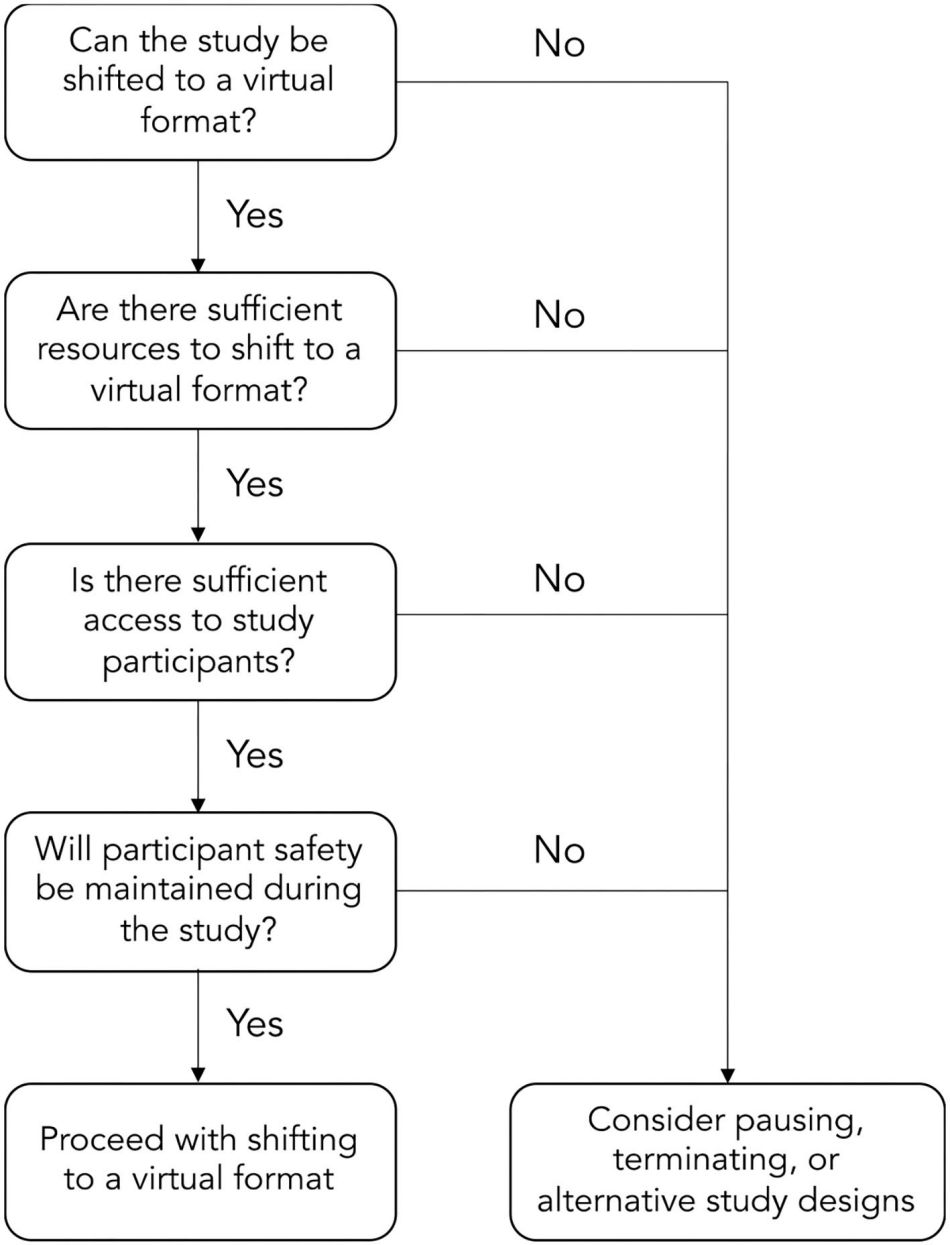
Potential flow of decisions to shift to a virtually-delivered rehabilitation intervention.

When recruitment is not possible, some researchers were faced with the inevitable reality of having to terminate or temporarily suspend studies. By consequence, pivoting a research program is often necessary. During to the COVID-19 pandemic, researchers have not always been able to collect pilot data to inform larger-scale trials. Alternative study methods have been employed such as secondary data analyses, accessing longitudinal datasets, and preparing systematic reviews. These projects allow trainees and early-career researchers to maintain research productivity, rationalize their future research, and provide learning opportunities during the COVID-19 pandemic. Nonetheless, the long-term implications of altering the direction of learning objectives and research programs will be an important consideration for both trainees and faculty. A potential flow of decisions for rehabilitation researchers to consider when shifting to a virtual format are depicted in the Figure 1.

### PART 3: Knowledge Dissemination

#### Challenge #1: Should I Plan to Attend Virtual Conferences?

The COVID-19 pandemic has provided researchers with a unique opportunity to establish and strengthen new and existing partnerships in a virtual environment. However, virtual conferences have been met with several shortcomings. Some trainees and faculty felt disconnected from the presenters and required additional preparation time and resources for this format. For instance, high-quality video and voice recordings are often needed to transition from in-person conferences to a virtual format since many conferences pre-record sessions to increase accessibility. Conference organizers have also explored several different online platforms for hosting conferences, requiring time consuming adaptation for researchers (16). Perhaps the greatest limitation of virtual conferences is a lack of in-person interactions and networking and sharing research. Despite improved accessibility in the virtual format, researchers agreed that these limitations contribute to reduced engagement, and offer a more limited experience.

##### Proposed Strategies

Participants suggested that conference organizers implement creative approaches to virtual conferences, such as small-group networking events to facilitate active and engaged knowledge dissemination with trainees and faculty from different institutions. Researchers in the workshop previously attended small portions of virtual conferences, and found smaller, more intimate networking sessions helped provide a supplementary and informal opportunity for rehabilitation researchers to connect. Promoting an environment that is supportive of spontaneous discussion about research topics will be vitally important as we move forward.

#### Challenge #2: What Are the Implications for My Study's Findings?

The publication process is an important aspect of knowledge dissemination that has been impacted by the COVID-19 pandemic (16). Rehabilitation science often involves the study of human behavior, experiences, characteristics, and traits such as physical activity, socioeconomic status and psychosocial factors. Each of these factors can be substantially altered during times of social isolation (17, 18). Hence, there is an increased importance of contextualizing research findings during a global pandemic. Participants included in virtual trials may not be representative of the population of interest, since accessibility to technology is not uniform across socioeconomic class and ethnicity (19). Finally, researchers identified a prolonged peer-review process for non-COVID-19 manuscript submissions, which impacts the timeliness in disseminating research findings to knowledge users and other researchers.

##### Proposed Strategies

Researchers suggested including information in the study's results and discussion sections to allow consideration of the unique influence of the pandemic on a study's outcomes and sample. To account for delays in peer-review, researchers recommended careful consideration in knowledge translation plans for grant applications and project monitoring. Increased requests for journal reviews may also offer additional opportunities for trainees seeking this experience.

## Discussion

The COVID-19 pandemic has changed rehabilitation research in many ways. We have proposed several challenges together with viable strategies to conducting research in this era. While some in-patient rehabilitation research has continued (20), virtually-delivered research have become the preferred and necessary alternative in community settings to ensure that study staff and participants follow public health guidelines and remain physically distant.

However, several challenges remain. *Pre-planned* virtually-delivered rehabilitation interventions are both feasible and effective in many patient populations (8, 21–23), but there is limited evidence on the effectiveness of shifting rehabilitation from in-person to virtual formats. Moreover, there is currently limited evidence about the psychometric properties (e.g., validity, reliability, and responsiveness) of remotely administered rehabilitation outcomes, which may have serious methodological implications for trials, such as estimating sample sizes and outcome selection. Finally, although this was not directly addressed in the workshop discussion, the COVID-19 pandemic is likely to alter timelines for trainees completing their theses and dissertations. Additional consideration should be given to trainees' timelines for program and study completion. Overall, there exists an excellent opportunity for rehabilitation researchers to address the gaps in virtually delivered rehabilitation research studies, as well as how to respond to trainees needs (e.g., networking opportunities, program completion) as we move forward in the pandemic.

## Strengths and Limitations

We acknowledge the strengths and limitations to our article. The challenges and proposed solutions discussed in this paper are based on the perspective of faculty and trainees from a single university institution in the Canadian context. It is possible that some of the discussion may be less directly relevant to researchers in other countries or institutions. However, since it is a global pandemic many rehabilitation researchers will be addressing similar issues. We also posit that rehabilitation researchers outside our specific context may adopt some of the strategies provided to fit their specific context. Our perspectives add to the existing literature on strategies for continuing research during a global pandemic. We also recognize that, while our intention was to include diverse perspectives from different areas of rehabilitation science, those of speech language pathologists or research staff were not captured. Moreover, it was not possible to disaggregate our findings by sex, gender, or other intersectional perspectives. Future research is needed to better understand these issues. As a perspective article, we welcome commentary and dialogue from other institutions on this issue, as other groups are likely to provide meaningful insight. We also acknowledge that the workshop was held in late 2020, and that this paper was written in 2021, and it is likely that the rehabilitation research landscape has evolved, even within our own institution. However, an important strength of our discussion is that the majority of challenges and potential solutions we have presented remain applicable for current and future global events, as the pandemic and modifications to work-life continue.

## Conclusion

This perspective paper addresses many current challenges experienced by rehabilitation researchers in the COVID-19 pandemic. There exist many strategies to pivoting, planning, and presenting rehabilitation research within the context of the COVID-19 pandemic. The proposed strategies in this article may help successfully advance some rehabilitation research programs during a global pandemic. However, researchers should carefully consider implications of these changes and contextualize their findings to the pandemic.

## Data Availability Statement

The original contributions presented in the study are included in the article/supplementary material, further inquiries can be directed to the corresponding author/s.

## Author Contributions

KSN and JR conceived the article. KSN contributed to data collection, interpretation, and led the manuscript preparation. LN, AM, and EW contributed to data collection and contributed to manuscript preparation. SS, KM, JCR, NB, LG, JV, CD'A, AK, ZL, EK, MKB, LM, BV, SEM, LCC, LJL, MEK, and JR contributed to manuscript preparation. All authors contributed to the article and approved the submitted version.

## Conflict of Interest

The authors declare that the research was conducted in the absence of any commercial or financial relationships that could be construed as a potential conflict of interest.

## Publisher's Note

All claims expressed in this article are solely those of the authors and do not necessarily represent those of their affiliated organizations, or those of the publisher, the editors and the reviewers. Any product that may be evaluated in this article, or claim that may be made by its manufacturer, is not guaranteed or endorsed by the publisher.

## References

[1] Polack FP Thomas SJ Kitchin N Absalon J Gurtman A Lockhart S. Safety and efficacy of the BNT162b2 mRNA Covid-19 vaccine. N Engl J Med. (2020) 383:2603–15. 10.1056/NEJMoa2034577 33301246 PMC7745181

[2] Baden LR El Sahly HM Essink B Kotloff K Frey S Novak R. Efficacy and safety of the mRNA-1273 SARS-CoV-2 vaccine. N Engl J Med. (2021) 384:403–16. 10.1056/NEJMoa2035389 33378609 PMC7787219

[3] Sadoff J Le Gars M Shukarev G Heerwegh D Truyers C De Groot AM. Interim results of a phase 1–2a trial of Ad26.COV2.S Covid-19 vaccine. N Engl J Med. (2021) 384:1824–35. 10.1056/NEJMoa2034201 33440088 PMC7821985

[4] Sohrabi C Mathew G Franchi T Kerwan A Griffin M Soleil C Del Mundo J. Impact of the coronavirus (COVID-19) pandemic on scientific research and implications for clinical academic training – A review. Int J Surg. (2021) 86:57–63. 10.1016/j.ijsu.2020.12.008 33444873 PMC7833269

[5] Cook DJ Kho ME Duan EH Alhazzani W Takaoka A Clarke FJ. Principles guiding nonpandemic critical care research during a pandemic. Crit Care Med. (2020) 48:1403–10. 10.1097/CCM.0000000000004538 32796181 PMC7437406

[6] Gill SV Shin D Ayoub M Keegan L Desrochers PC Helfrich CA. Pivoting in context: using the forging alliances in interdisciplinary rehabilitation research model to collaborate during COVID-19. Am J Phys Med Rehabil. (2021) 100:519–25. 10.1097/PHM.0000000000001749 33782276 PMC8131232

[7] Maguire R Hynes S Seebacher B Block VJ Zackowski KM Jonsdottir J. Research interrupted: the impact of theCOVID-19 pandemic on multiple sclerosisresearch in the field of rehabilitation andquality of life. Multiple Scler J Exp Transl Clin. (2021) 7:1–6. 10.1177/20552173211038030 34471543 PMC8404642

[8] Lavoie V Bouchard M Turcotte S Tousignant M. Telerehabilitation for individuals with Parkinson's disease and a history of falls: a pilot study. Physiother Can. (2021) 73:e20190108. 10.3138/ptc-2019-0108 34880539 PMC8614589

[9] Saladino V Algeri D Auriemma V. The psychological and social impact of Covid-19: new perspectives of well-being. Front Psychol. (2020). 11:577684. 10.3389/fpsyg.2020.577684 33132986 PMC7561673

[10] Marzolini S Ghisi GLDM Hébert A-A Ahden S Oh P. Cardiac rehabilitation in Canada during COVID-19. CJC Open. (2021) 3:152–8. 10.1016/j.cjco.2020.09.021 33521613 PMC7833488

[11] Jordan RE Adab P Cheng KK. Covid-19: risk factors for severe disease and death. BMJ. (2020) 368:m1198. 10.1136/bmj.m1198 32217618

[12] Sepúlveda-Loyola W Rodríguez-Sánchez I Pérez-Rodríguez P Ganz F Torralba R Oliveira DV. Impact of social isolation due to COVID-19 on health in older people: mental and physical effects and recommendations. J Nutr Health Aging. (2020) 24:938–47. 10.1007/s12603-020-1500-7 33155618 PMC7597423

[13] Fenerty SD West C Davis SA Kaplan SG Feldman SR. The effect of reminder systems on patients' adherence to treatment. Patient Prefer Adher. (2012) 127. 10.2147/PPA.S26314 PMC3287416 22379363

[14] Centers for Disease Control Prevention. COVID Data Tracker. Atlanta, GA: US Department of Health and Human Services, CDC (2022). Available online at: https://covid.cdc.gov/covid-data-tracker

[15] COVIDSurg Collaborative. Elective surgery cancellations due to the COVID-19 pandemic: global predictive modelling to inform surgical recovery plans. Br J Surg. (2020) 107:1440–9. 10.1002/bjs.11746 32395848 PMC7272903

[16] Bernard MA Lauer M. The Impact of the COVID-19 Pandemic on the Extramural Scientific Workforce – Outcomes from an NIH-Led Survey. NIH Office of Extramural Research (2021). Available online at: https://nexus.od.nih.gov/all/2021/03/25/the-impact-of-the-covid-19-pandemic-on-the-extramural-scientific-workforce-outcomes-from-an-nih-led-survey/ (accessed May 17, 2021).

[17] Peçanha T Goessler KF Roschel H Gualano B. Social isolation during the COVID-19 pandemic can increase physical inactivity and the global burden of cardiovascular disease. Am J Physiol Heart Circ Physiol. (2020) 318:H1441–6. 10.1152/ajpheart.00268.2020 32412779 PMC7303725

[18] Robb CE De Jager CA Ahmadi-Abhari S Giannakopoulou P Udeh-Momoh C Mckeand J. associations of social isolation with anxiety and depression during the early COVID-19 pandemic: a survey of older adults in London, UK. Front Psychiatry. (2020) 11:591120. 10.3389/fpsyt.2020.591120 33132942 PMC7566017

[19] Yardi S Bruckman A. Income, race, and class: exploring socioeconomic differences in family technology use. In: Proceedings of the SIGCHI Conference on Human Factors in Computing Systems. Austin, TX (2012), 3041−50.

[20] Kho ME Molloy AJ Clarke FJ Reid JC Herridge MS Karachi T. Multicentre pilot randomised clinical trial of early in-bed cycle ergometry with ventilated patients. BMJ Open Respiratory Res. (2019) 6:e000383. 10.1136/bmjresp-2018-000383 30956804 PMC6424272

[21] Camden C Pratte G Fallon F Couture M Berbari J Tousignant M. Diversity of practices in telerehabilitation for children with disabilities and effective intervention characteristics: results from a systematic review. Disabil Rehabil. (2020) 42:3424–36. 10.1080/09638288.2019.1595750 30978110

[22] Dias JF Oliveira VC Borges PRT Dutra FCMS Mancini MC Kirkwood RN. Effectiveness of exercises by telerehabilitation on pain, physical function and quality of life in people with physical disabilities: a systematic review of randomised controlled trials with GRADE recommendations. Br J Sports Med. (2020) 55:155–62. 10.1136/bjsports-2019-101375 33060156

[23] Scherrenberg M Falter M Dendale P. Cost-effectiveness of cardiac telerehabilitation in coronary artery disease and heart failure patients: systematic review of randomized controlled trials. Eur Heart J Digital Health. (2020) 1:20–9. 10.1093/ehjdh/ztaa005 PMC10087016 37056294

